# Genomic sequences of free-living *Pseudoalteromonas* species isolated from seawater using an ethylene-α-olefin co-oligomer as a sole carbon source

**DOI:** 10.1128/mra.00785-25

**Published:** 2025-08-29

**Authors:** Ryo Iizuka, Takao Yoshida, Masaru Kawato, Yoshihiro Fujiwara, Sotaro Uemura

**Affiliations:** 1 Department of Biological Sciences, Graduate School of Science, The University of Tokyo 13143, Tokyo, Japan; 2 Japan Agency for Marine-Earth Science and Technology (JAMSTEC) 13570, Yokosuka, Japan; 3 Core Research for Evolutional Science and Technology (CREST), Japan Science and Technology Agency 13501, Tokyo, Japan; Montana State University, Bozeman, Montana, USA

**Keywords:** *Pseudoalteromonas*, ethylene-α-olefin co-oligomer, polyolefin

## Abstract

Two free-living *Pseudoalteromonas* species were isolated from surface seawater through enrichment cultures using a model compound of polyolefins as the sole carbon source. We report their complete and draft genomes. Although these genomes lack genes encoding alkane hydroxylases involved in polyolefin degradation, they provide insights into polyolefin-bacteria interactions.

## ANNOUNCEMENT

*Pseudoalteromonas* species are prevalent in marine environments, with some known for attaching to hydrocarbons and plastics (1–4), suggesting their potential involvement in the processing of these materials. We present genomic characterization of *Pseudoalteromonas* species isolated using an ethylene-α-olefin co-oligomer (LUCANT HC-40, Mitsui Chemicals), providing insights into microbial-polyolefin interactions in marine environments.

Surface seawater was collected from Suruga Bay, Japan (34°44.93′N, 138°40.41′E) on 24 April 2024, and off Minamidaito Island, Japan (25°50.75′N, 131°22.69′E) on 3 May 2024, during the R/V *Kaimei* research cruise (KM24-03 Leg 1 and 2). The seawater was concentrated ∼1,000-fold by ultrafiltration (5) and inoculated into artificial seawater containing 0.1% NH_4_Cl and 1% LUCANT HC-40. After 20 days at 4°C, a small portion of the LUCANT material was streaked onto an agar plate containing artificial seawater medium (ASM) (6–8) and incubated for 20 days at 4°C. This process yielded strain 6L1 from seawater collected at Suruga Bay and strain 11L1 from seawater collected off Minamidaito Island. 16S rRNA gene analysis (7, 8) identified them as members of the *Pseudoalteromonas* genus. Prior to genomic sequencing, the strains were cultured aerobically in ASM for 27 h at 10°C. Genomic DNA was extracted using Quick-DNA Fungal/Bacterial Miniprep Kit (Zymo Research). The DNA was not sheared or size-selected prior to library preparation. SMRTbell libraries were prepared using SMRTbell prep kit 3.0 (PacBio) and then bound to DNA polymerase using Revio Polymerase Kit (PacBio). Sequencing was performed using the Revio system (PacBio). Flye (v2.9.4) (9) was used to assemble raw sequencing reads directly without quality preprocessing (adapter trimming, length/quality filtering, or error correction), with the options --asm-coverage 200 --genome-size 4m, and automatically eliminated overlapping ends to generate circular contigs. DFAST (v1.6.0) (10–12) was used for taxonomic classification, including average nucleotide identity (ANI) calculation, genome quality assessment, and annotation. Default parameters were used except where otherwise noted.

The 6L1 genome was assembled into two linear contigs and a circular contig, presumably representing the primary replicon (chromosome) fragments and a secondary replicon (chromid), respectively (Table 1). The closest relative was *Pseudoalteromonas marina* DSM 17587^T^ (GCF_000238335.3) (13), with an ANI of 97.90%, representing a new *P. marina* strain. The 11L1 genome comprised two circular contigs, corresponding to a chromosome and a chromid (Table 1). This genome was closely related to that of *Pseudoalteromonas undina* DSM 6065^T^ (GCF_000238275.3) (14, 15), with an ANI of 96.26%, suggesting a novel *P. undina* strain. Both genomes encode exopolysaccharide biosynthesis and export proteins, as well as multicopper oxidases, which may contribute to polyolefin interactions and fragmentation (16, 17). However, both genomes lack genes encoding alkane hydroxylases, typically involved in the aerobic degradation of polyolefin fragments (18, 19), suggesting that these bacteria may survive on polyolefin surfaces through novel mechanisms beyond canonical degradation pathways. These findings expand our understanding of microbial strategies for colonizing synthetic polymer surfaces in marine environments.

**TABLE 1:** Sequencing statistics, genomic features, and accession numbers of strains 6L1 and 11L1

	6 L1	11 L1
BioSample accession number	SAMD00952982	SAMD00952983
Isolation source	Surface seawater from Suruga Bay	Surface seawater off Minamidaito Island
Sequencing results		
Number of reads	354,971	4,41,839
Sum of length (bp)	2,734,544,880	3,522,735,282
Read N50 (bp)	10,142	9,648
SRA accession number	DRR689906	DRR689907
Assembly results		
Assembly status	Draft	Complete
Length (bp)		
Contig 1	3,732,983 (Linear)	3,379,871 (Circular)
Contig 2	728,500 (Circular)^*a*^	749,062 (Circular)^*a*^
Contig 3	12,444 (Linear)	–^*b*^
GC content (%)		
Contig 1	40	40.2
Contig 2	39.8	39.5
Contig 3	41.4	–
Mean coverage (×)	611	853
Annotation results		
Number of protein-coding sequences		
Contig 1	3,386	3,047
Contig 2	658	658
Contig 3	12	–
Number of rRNAs		
Contig 1	25	25
Contig 2	3	3
Contig 3	0	–
Number of tRNAs		
Contig 1	103	102
Contig 2	0	0
Contig 3	0	–
Completeness check		
Completeness (%)	100	100
Contamination (%)	0.53	0.51
GenBank accession number		
Contig 1	BAAHWQ010000001	AP041141
Contig 2	LC883538	AP041142
Contig 3	BAAHWQ010000002	–

^*a*^ Both strains have small circular contigs (~0.73 Mb for 6L1 and ~0.75 Mb for 11L1) that contain rRNA genes. These contigs also include a small proportion of single-copy essential marker genes used in CheckM analysis (20), with 8.08% for 6L1 and 8.33% for 11L1. These contigs likely function as chromids, which are commonly found in *Pseudoalteromonas* species (0.6–1.8 Mb) (21–24).

^*b*^ "–”, not applicable.

## Data Availability

Strains 6L1 and 11L1 are associated with BioProject accession number PRJDB21836, and the accession numbers are listed in Table 1.
